# Lifestyle Factors Associated with Type 2 Diabetes and Use of Different Glucose-Lowering Drugs: Cross-Sectional Study

**DOI:** 10.1371/journal.pone.0111849

**Published:** 2014-11-04

**Authors:** Sinna P. Ulrichsen, Anil Mor, Elisabeth Svensson, Finn B. Larsen, Reimar W. Thomsen

**Affiliations:** 1 Department of Clinical Epidemiology, Faculty of Health, Aarhus University Hospital, Aarhus, Denmark; 2 Centre for Public Health and Quality Improvement, Central Denmark Region, Aarhus, Denmark; Virgen Macarena University Hospital, School of Medicine, University of Seville, Spain

## Abstract

**Aims:**

To examine the lifestyle profile among persons with and without Type 2 diabetes mellitus (DM) and among users of different glucose-lowering drugs.

**Methods:**

We used questionnaire data from a Danish health survey and identified presence of Type 2 DM and use of medications through medical databases. We calculated age- and gender-standardized prevalence ratios (PRs) of lifestyle factors according to Type 2 DM and different glucose-lowering drugs.

**Results:**

Of 21,637 survey participants aged 25–79 years, 680 (3%) had Type 2 DM (median age 63 years) with a median diabetes duration of 5 years. Participants with Type 2 DM had a substantially higher prevalence of obesity (36% vs. 13%, PR: 3.1, 95% confidence interval (CI): 2.8–3.6), yet more reported to eat a very healthy diet (25% vs. 21%, PR: 1.2, 95% CI: 1.0–1.4) and to exercise regularly (67% vs. 53%, PR: 1.3, 95% CI: 1.2–1.4). Also, fewer were current smokers or had high alcohol intake. When compared with metformin users, obesity was substantially less prevalent in users of sulfonylurea (PR: 0.5, 95% CI: 0.4–0-8), and insulin and analogues (PR: 0.4, 95% CI: 0.3–0.7). Tobacco smoking was more prevalent in sulfonylurea users (PR: 1.4, 95% CI: 0.9–2.1) compared with metformin users. We found no material differences in physical exercise, diet or alcohol intake according to type of glucose-lowering drug.

**Conclusions:**

Type 2 DM patients are substantially more obese than other individuals, but otherwise report to have a healthier lifestyle. Metformin use is strongly associated with obesity, whereas sulfonylurea use tends to be associated with tobacco smoking.

## Introduction

Type 2 diabetes mellitus (DM) and its complications are an increasing challenge to health care systems worldwide [1], [2]. Diabetes confers a 1.8-fold increased risk of death from any cause, including a 2.3-fold increased risk of death from vascular causes, but also markedly increased risk of death from renal disease, liver disease, infections, mental disorders, and cancer [3]–[7]. Much of our knowledge on these associations is based on large observational registry-based studies, lacking information on lifestyle factors. Thus, it is unclear to which extent these associations (e.g. diabetes and increased risk of cancer) are related to unhealthier lifestyle in diabetes [6]–[8]. Similarly, comparative effectiveness studies of different glucose-lowering therapies in diabetes, as well as studies of important side effects or beneficial pleiotropic effects of these therapies, often rely on observational designs that also may be hampered by uncontrolled confounding by lifestyle factors [9]. Even in prospective studies with primary data collection, potential confounding often persists owing to unmeasured data on lifestyle factors, such as details about dietary habits and physical activity, associated with Type 2 DM [3]. Sensitivity analysis and external adjustment for unmeasured confounding by lifestyle factors allow for bias assessment, and therefore, detailed information about lifestyle behaviour associated with Type 2 DM and its treatment is needed [10].

We therefore aimed to examine lifestyle differences among persons with and without Type 2 DM and among users of different glucose-lowering drugs based on data from a Danish public health survey.

## Materials and Methods

### Setting

We conducted this population-based cross-sectional study in Central Denmark region, encompassing about 1.2 million inhabitants. Denmark is a welfare state with tax-funded universal access to health care, providing primary and secondary care without out-of-pocket expenses and partial reimbursement for most prescribed medications, including glucose-lowering drugs [11]. Individual-level data from all Danish registries can be linked via a unique personal identifier, the CPR number, assigned at birth and registered in the Danish Civil Registration system [12].

### Study population

**Figure 1:**
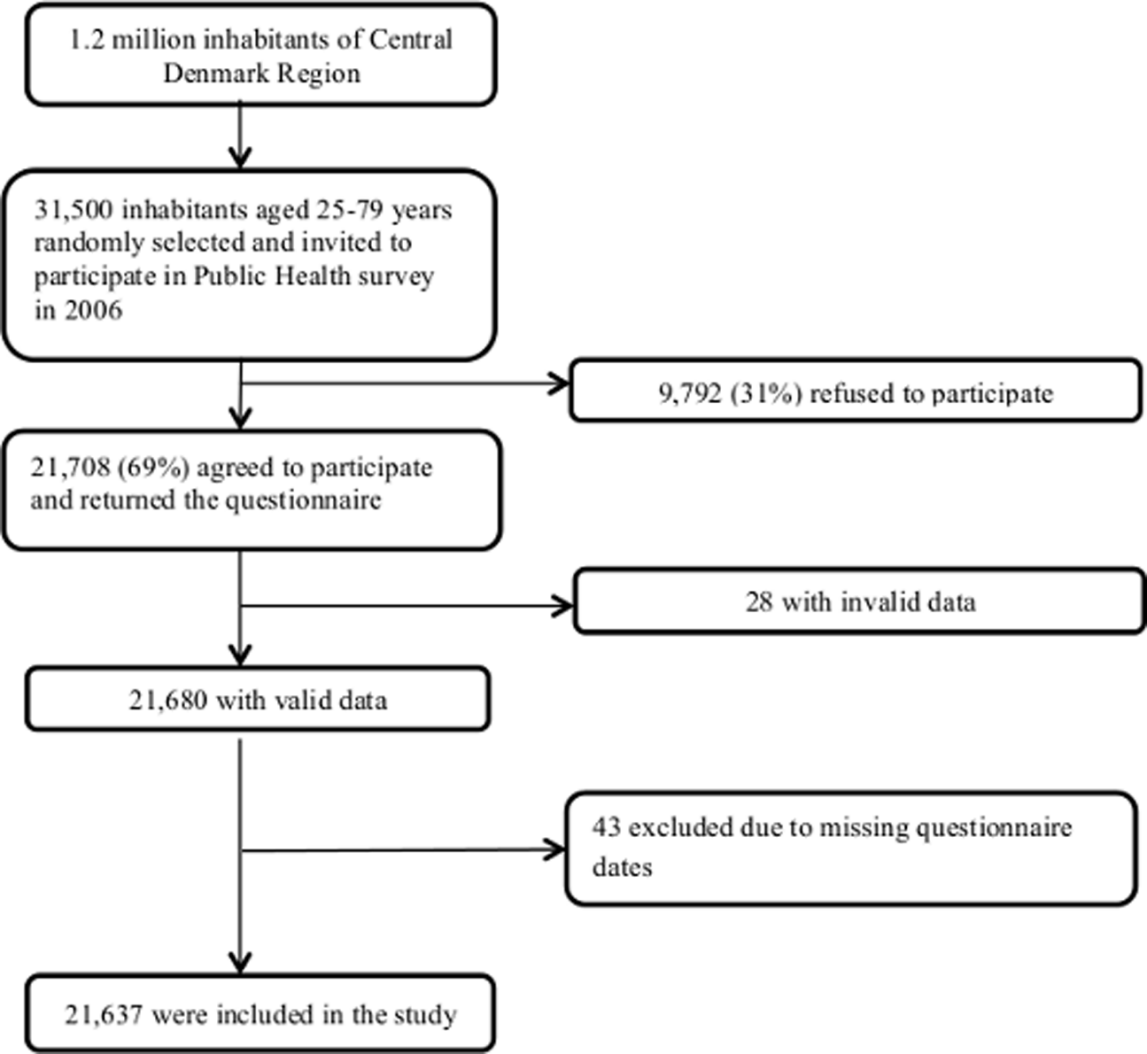
Selection of participants for the survey.

In 2006, the Central Denmark Region conducted a public health survey called “*Hvordan har du det*?” (English: “How are you?”). This region consists of 19 municipalities. The authorities decided to randomly select and invite 1500 participants from each municipality, except for one municipality encompassing the capital city of the region where 4500 participants were selected. A total of 31,500 Danish citizens between 25 and up to 79 years of age, living in the Central Denmark Region with at least one parent born in Denmark, were selected and invited to participate in the survey. In total, 21,708 (69%) invited persons agreed and gave their informed consent to participate in the survey. Following this, a detailed questionnaire with approximately 400 questions on self-rated health, occurrence of chronic diseases, socioeconomic factors, and lifestyle factors were sent to all respondents who agreed to participate. Furthermore, three reminders were sent to those who did not respond to the questionnaire to increase the response rate [13]. Finally, 21,637 participants returned valid questionnaire data. Figure 1 shows the selection process of the study.

For our present analyses based on the “How are you” data, approval by the Danish Scientific Ethical Committee was not needed according to Danish legislation, as our study was registry-based and did not include human biological material. The questionnaire was prepared in the Danish language and the study design, including the questionnaire and sampling method, has been described in more detail elsewhere [11], [14].

### Data on lifestyle factors

Lifestyle factors included in the questionnaire were body mass index (BMI), physical activity, diet, smoking status, and alcohol intake. BMI (kg/m^2^) was categorized according to the World Health Organization's (WHO) criteria as underweight (<18.5), normal weight (18.5–24.9), overweight (25.0–29.9) and obese (≥30.0) [15]. Regular physical exercise was defined as the participation in leisure sports or other regular physical exercise (yes, no). Based on 30 detailed questions about fruit, vegetables, fish, and fat intake, a validated diet score was calculated, and categorized into very healthy (high amount of fruit, vegetables, fish, and low amount of saturated fat), reasonably healthy (moderately high intake of fruit, vegetables, fish, and saturated fat), or unhealthy (low intake of fruit, vegetables, fish, and high amount of saturated fat) [16]. Smoking status was defined as current (daily or occasionally), former, or never tobacco smoking. Alcohol intake was categorized as above or within the recommended maximum intake at the time of the survey (≤14/≤21 weekly drinks for women/men, respectively).

### Patients with Type 2 DM

We defined Type 2 DM as persons who were at least 30 years of age at the time of first hospital contact with diabetes or who received any oral glucose-lowering drug at any time. Diabetes duration was defined as years between the first diabetes diagnosis or drug prescription and the date of filling the questionnaire. We identified all individuals with any diabetes-related hospital contact in the Danish National Registry of Patients (DNRP) (see codes in the appendix S1), and identified glucose-lowering drug prescription using Anatomical Therapeutic Chemical (ATC) codes in the Aarhus University Prescription Database (AUPD) (see the appendix S1 for ATC codes). The DNRP contains complete hospitalization history of all Danish residents since 1977 with one primary discharge diagnosis and up to 20 secondary discharge diagnosis coded according to the International Classification of Diseases (ICD) 8^th^ revision (ICD-8) until the end of 1993, and 10^th^ revision (ICD-10) thereafter [17]. The AUPD include information on all reimbursed prescriptions since 1996 from former Aarhus County, and since 1998 from former Viborg and Ringkjøbing Counties [18].

### Glucose-lowering drugs

We categorized Type 2 DM patients according to filled glucose-lowering drug prescriptions in the AUPD within 100 days before and 100 days after the date of returning the questionnaire. This period was chosen to capture most current users of glucose-lowering drugs, as most glucose-lowering drug prescriptions are expected to last for 90 or 100 days in Denmark. Patients reimbursing only one type of glucose-lowering drug were categorized according to the following categories (codes in the appendix S1): metformin, sulfonylureas, insulin and insulin analogues, and other glucose-lowering drugs. Patients with prescriptions for more than one of the above types of glucose-lowering drugs or with combination tablets were categorized as combination users. Patients not filling any prescription within the 200 days were categorized as Type 2 DM patients with no medical treatment.

### Data on comorbidity

Based on hospital diagnosis codes from the DNRP we computed the Charlson comorbidity index (CCI) score for each person defining three comorbidity levels: low (CCI score of 0), medium (CCI score of 1–2), and high (CCI score of 3+) [19]. The two diabetes categories were left out of the CCI score as diabetes defined our index disease [20].

### Statistical analyses

We estimated the prevalence of different lifestyle factors, demographic variables, and comorbidity level according to Type 2 DM status and use of different glucose-lowering drugs. We calculated age- and gender-standardized prevalence differences (PDs) and prevalence ratios (PRs) for lifestyle factors, comparing Type 2 DM patients with other survey participants standardized to the cohort of all participants, and comparing users of different glucose-lowering drugs standardized to the cohort of Type 2 DM patients. For the analysis of glucose-lowering drug users, we used metformin monotherapy as reference. We repeated the analyses with restriction to newly diagnosed Type 2 DM patients, i.e. those who had been diagnosed within three years of the survey.

All statistical analyses were performed using Statistical Analysis System (SAS) software (version 9.2, SAS Institute, Cary, NC). This project was approved by the Danish Data Protection Agency (record number 2013-41-1924).

## Results

A total of 680 (3.1%) of the 21,637 persons aged between 25 and 80 years were identified as having Type 2 DM. Regarding glucose-lowering drugs, 16% (106/680) patients were treated with metformin, 28% (191/680) with combination therapy, 14% (94/680) with sulfonylureas, 17% (113/680) with insulin and analogues, <1% (4/680) with other glucose-lowering drugs (all were repaglinide users), and 25% (172/680) received no glucose-lowering drugs. Among the 191 combination users, 180 (94%) were treated with metformin in combination, 118 (66%) with sulfonylureas in combination, and 73 (38%) with insulin and analogues in combination.

### Patient characteristics

Type 2 DM patients were older than other survey participants (median age 63 years (interquartile range (IQR), 56–70 years) vs 51 years (IQR, 40–62 years)) (*Table 1*). The median duration of diabetes in the Type 2 DM population was 5 years (IQR, 2–10 years) and 36% had a history of hospital-diagnosed comorbidity included in the CCI, compared with 13% of the other survey participants.

**Table 1:** Characteristics of 21,637 participants according to type 2 diabetes and glucose-lowering drug use.

	Type 2 diabetes		Glucose-lowering drugs					
	No	Yes	Metformin	Combination*	Sulfonylureas	Insulin and analogues	Other glucose-lowering drugs	No glucose-lowering drug
	*n = 20,957* (%)	*n = 680* (%)	*n = 106* (%)	*n* = 191 (%)	*n* = 94 (%)	*n* = 113 (%)	*n* = 4 (%)	*n* = 172 (%)
**Gender**								
Women	11172 (53)	336 (49)	56 (53)	97 (51)	46 (49)	47 (42)	2 (50)	88 (51)
Men	9785 (47)	344 (51)	50 (47)	94 (49)	48 (51)	66 (58)	2 (50)	84 (49)
**Age, median (IQR)**	51 (40, 62)	63 (56, 70)	65 (57, 69)	62 (54, 70)	68 (60, 73)	61 (55, 71)	61 (53, 65)	62 (55, 70)
<45 years	7585 (36)	56 (8)	12 (11)	14 (7)	1 (1)	10 (9)	0 (0)	19 (11)
45–64 years	9434 (45)	312 (46)	38 (36)	98 (51)	35 (37)	56 (50)	3 (75)	82 (48)
65–80 years	3938 (19)	312 (46)	56 (53)	79 (41)	58 (62)	47 (42)	1 (25)	71 (41)
**Diabetes duration, median (IQR)**	–	5 (2, 10)	3 (1, 5)	7 (4, 9)	4 (2, 7)	12 (8, 18)	6 (3, 9)	4 (2, 8)
0–2 years	–	204 (30)	52 (49)	35 (18)	36 (38)	11 (10)	1 (25)	69 (40)
3–5 years	–	154 (23)	32 (30)	43 (23)	26 (28)	12 (11)	1 (25)	41 (24)
6–10 years	–	192 (28)	17 (16)	89 (47)	25 (27)	27 (24)	2 (50)	34 (20)
11–15 years	–	66 (10)	5 (5)	15 (8)	5 (5)	29 (26)	0 (0)	12 (7)
16–20 years	–	38 (6)	0 (0)	6 (3)	2 (2)	19 (17)	0 (0)	11 (6)
21–30 years	–	23 (3)	0 (0)	3 (2)	0 (0)	15 (13)	0 (0)	5 (3)
**CCI score**								
0	18292 (87)	434 (64)	76 (72)	137 (72)	60 (64)	67 (59)	3 (75)	91 (53)
1–2	2355 (11)	198 (29)	25 (24)	47 (25)	23 (24)	33 (29)	1 (25)	69 (40)
3+	310 (2)	48 (7)	5 (5)	7 (4)	11 (12)	13 (12)	0 (0)	12 (7)
**Myocardial infarction**	286 (1)	46 (7)	6 (6)	3 (2)	6 (6)	12 (11)	1 (25)	18 (10)
**Congestive heart failure**	147 (1)	45 (7)	3 (3)	8 (4)	11 (12)	12 (11)	0 (0)	11 (6)

CCI =  Charlson comorbidity index, IQR =  inter-quartile range.

*Patients using more than one type of glucose-lowering drug or using combination tablets.

*Table 1* shows demographic and clinical characteristics according to glucose-lowering drug groups. Sulfonylurea users tended to be older compared with users of any other glucose-lowering drugs. Users of insulin and analogues had longest diabetes history (median duration 12 years, IQR, 8–18), followed by combination therapy users (7 years, IQR, 4–9). Metformin users had a comparably short history of diabetes (median duration 3 years, IQR, 1–5), and also had lower prevalence of comorbidities as compared with other drug groups.

### Lifestyle behavior according to Type 2 DM status

*Table 2* shows the distribution of lifestyle factors according to diabetes status. Compared with other survey participants, a substantially higher proportion of individuals with Type 2 DM were obese (36% vs. 13%, PR: 3.1, 95% CI: 2.8–3.6). However, more reported eating a very healthy diet (25% vs. 21%, PR: 1.2, 95% CI: 1.0–1.4), and more were engaged in regular physical exercise (67% vs. 53%, PR: 1.3, 95% CI: 1.2–1.4). Type 2 DM patients were also less likely to be current smokers (24% vs. 29%, PR: 0.9, 95% CI: 0.8–1.1) and a slightly lower proportion had alcohol intake over the recommended limits (5% vs. 6%, PR: 0.7, 95% CI: 0.5–1.1) (*Table 2*). After restricting the analyses to newly diagnosed Type 2 DM patients, we did not find any material difference from the complete cohort estimates (*Table 3*).

**Table 2:** Age-and gender-standardized prevalence differences (PDs) and prevalence ratios (PRs) of lifestyle factors according to type 2 diabetes.

	Type 2 diabetes		PDs comparing T2DM patients vs. other individuals	PRs comparing T2DM patients vs. other individuals
	No	Yes		
	*n* = 20,957 (%)	*n = 680* (%)	PDs (95% CIs)	PRs (95% CIs)
**BMI (kg/m^2^)**				
<18.5	308 (1)	4 (1)	−1.0 (−1.5 to −0.5)	0.3 (0.1 to 0.9)
18.5–24	9979 (48)	163 (24)	−23.8 (−28.3 to −19.3)	0.5 (0.4 to 0.6)
25–29	7490 (36)	234 (34)	−4.8 (−9.5 to 0.0)	0.9 (0.7 to 1.0)
≥30	2708 (13)	243 (36)	27.6 (22.6 to 32.6)	3.1 (2.8 to 3.6)
Missing	472 (2)	36 (5)	–	–
**Diet** *				
Unhealthy	2809 (13)	71 (10)	−1.3 (−5.1 to 2.6)	0.9 (0.7 to 1.3)
Reasonably healthy	13320 (64)	404 (59)	−3.1 (−8.4 to 2.2)	1.0 (0.9 to 1.0)
Very healthy	4363 (21)	170 (25)	3.4 (−1.0 to 7.8)	1.2 (1.0 to 1.4)
Missing	465 (2)	35 (5)	–	–
**Smoking**				
Current	6170 (29)	162 (24)	−1.8 (−6.7 to 3.2)	0.9 (0.8 to 1.1)
Former	5261 (25)	223 (33)	−0.7 (−4.6 to 3.2)	1.0 (0.8 to 1.1)
Never	8803 (42)	235 (35)	−0.6 (−5.9 to 4.7)	1.0 (0.9 to 1.1)
Missing	723 (3)	60 (9)	–	–
**Alcohol intake** †				
High	1234 (6)	33 (5)	−1.8 (−3.6 to 0.0)	0.7 (0.5 to 1.1)
Low	19211 (92)	620 (91)	0.6 (−2.1 to 3.2)	1.0 (1.0 to 1.0)
Missing	512 (2)	27 (4)	–	–
**Regular physical exercise** ‡				
Yes	11031 (53)	455 (67)	15.6 (10.7 to 20.6)	1.3 (1.2 to 1.4)
No	9489 (45)	200 (29)	−15.6 (−20.5 to −10.6)	0.7 (0.6 to 0.8)
Missing	437 (2)	25 (4)	–	–

T2DM =  type 2 diabetes mellitus, BMI =  body mass index, CI =  confidence interval.

*Diet: very healthy (high amount of fruit, vegetables, fish and low amount of saturated fat), reasonably healthy (moderately high amount of fruit, vegetables, fish and saturated fat) or unhealthy (low amount of fruit, vegetables, fish and high amount of saturated fat).

† Alcohol intake: High (above the recommended limit of ≤14/≤21 weekly drinks for women/men) or low (within the recommended limit).

‡ Regular exercise: participation in leisure sports or other regular physical activity (yes/no).

**Table 3:** Age-and gender-standardized prevalence differences (PDs) and prevalence ratios (PRs) of lifestyle factors among newly diagnosed^#^ type 2 diabetes patients.

	Type 2 diabetes		PDs comparing T2DM patients vs. other individuals	PRs comparing T2DM patients vs. other individuals
	No	Yes		
	*n = 20,957* (%)	*n = 204* (%)	PDs (95% CIs)	PRs (95% CIs)
**BMI (kg/m^2^)**				
<18.5	308 (1)	0 (0)	–	–
18.5–24	9979 (48)	40 (20)	−29.0 (−35.1 to −22.8)	0.4 (0.3 to 0.5)
25–29	7490 (36)	65 (32)	−5.0 (−12.5 to 2.4)	0.9 (0.7 to 1.1)
≥30	2708 (13)	83 (41)	31.0 (23.2 to 38.8)	3.4 (2.8 to 4.1)
Missing	472 (2)	16 (8)	–	–
**Diet** *				
Unhealthy	2809 (13)	22 (11)	−2.3 (−7.5 to 3.0)	0.8 (0.5 to 1.3)
Reasonably healthy	13320 (64)	118 (58)	−3.6 (−11.5 to 4.3)	0.9 (0.8 to 1.1)
Very healthy	4363 (21)	51 (25)	4.1 (−2.9 to 11.2)	1.2 (0.9 to 1.6)
Missing	465 (2)	13 (6)	–	–
**Smoking**				
Current	6170 (29)	51 (25)	−1.7 (−9.1 to 5.8)	0.9 (0.7 to 1.2)
Former	5261 (25)	77 (38)	6.1 (−0.9 to 13.2)	1.2 (1.0 to 1.6)
Never	8803 (42)	60 (29)	−7.9 (−15.7 to 0.0)	0.8 (0.7 to 1.1)
Missing	723 (3)	16 (8)	–	–
**Alcohol intake** †				
High	1234 (6)	12 (6)	−1.9 (−4.2 to 0.4)	0.7 (0.4 to 1.2)
Low	19211 (92)	184 (90)	1.1 (−2.4 to 4.6)	1.0 (1.0 to 1.1)
Missing	512 (2)	8 (4)	–	–
**Regular physical exercise** ‡				
Yes	11031 (53)	141 (69)	19.5 (12.7 to 26.4)	1.4 (1.3 to 1.5)
No	9489 (45)	57 (28)	−18.7 (−25.6 to −11.9)	0.6 (0.5 to 0.8)
Missing	437 (2)	6 (3)	–	–

T2DM =  type 2 diabetes mellitus, BMI =  body mass index, CI =  confidence interval.

#Newly diagnosed were all the patients diagnosed within 3 years of the survey date.

*Diet: very healthy (high amount of fruit, vegetables, fish and low amount of saturated fat), reasonably healthy (moderately high amount of fruit, vegetables, fish and saturated fat) or unhealthy (low amount of fruit, vegetables, fish and high amount of saturated fat).

† Alcohol intake: High (above the recommended limit of ≤14/≤21 weekly drinks for women/men) or low (within the recommended limit).

‡ Regular exercise: participation in leisure sports or other regular physical activity (yes/no).

### Lifestyle behavior according to glucose-lowering drug use

#### BMI

The crude prevalence of obesity ranged from 19% (insulin and analogues users) to 49% (metformin users). Patients using insulin and analogues were more likely (43%) to have a normal BMI (18.5–24 kg/m2) than any other drug users (*Table 4*). After standardizing for gender and age, the prevalence of obesity was clearly lower in users of sulfonylureas, insulin and analogues, and in those with no glucose-lowering drug use as compared with metformin users (PR: 0.5, 95% CI: 0.4–0-8; PR: 0.4, 95% CI: 0.3–0.7; PR: 0.6, 95% CI: 0.5–0.8, respectively). Prevalence differences for all medication categories as compared with metformin are shown in *Table 5*.

**Table 4:** Distribution of lifestyle factors according to glucose-lowering drug use among 680 individuals with type 2 diabetes participating in the Danish “How are you?” public health survey in 2006.

	Type 2 diabetes	Metformin	Combinationδ	Sulfonylureas	Insulin and analogues	No glucose-lowering drug
	*n = 680* (%)	*n = 106* (%)	*n = 191* (%)	*n = 94* (%)	*n = 113* (%)	*n = 172* (%)
**BMI (kg/m^2^)**						
<18.5	4 (1)	0 (0)	1 (1)	1 (1)	2 (2)	0 (0)
18.5–24	161 (24)	12 (11)	26 (14)	27 (29)	48 (43)	48 (28)
25–29	232 (34)	35 (33)	65 (34)	37 (39)	31 (27)	64 (37)
≥30	243 (36)	52 (49)	92 (48)	24 (26)	22 (19)	53 (31)
Missing	36 (5)	7 (7)	7 (4)	5 (5)	10 (9)	7 (4)
**Diet** *						
Unhealthy	71 (11)	11 (10)	15 (08)	10 (11)	14 (12)	21 (12)
Reasonably healthy	401 (59)	59 (56)	126 (66)	54 (57)	60 (53)	59 (59)
Very healthy	169 (25)	32 (30)	41 (21)	25 (27)	34 (30)	37 (22)
Missing	35 (5)	4 (4)	9 (5)	5 (5)	5 (4)	12 (7)
**Smoking**						
Current	160 (24)	23 (22)	36 (19)	28 (30)	29 (26)	44 (26)
Former	223 (33)	35 (33)	70 (37)	29 (31)	37 (33)	52 (30)
Never	233 (35)	40 (38)	64 (34)	30 (32)	40 (35)	59 (34)
Missing	60 (9)	8 (8)	21 (11)	7 (7)	7 (6)	17 (10)
**Alcohol intake** †						
High	32 (05)	7 (7)	6 (3)	4 (4)	7 (06)	8 (5)
Low	617 (91)	94 (89)	179 (94)	85 (90)	102 (90)	157 (91)
Missing	27 (4)	5 (5)	6 (3)	5 (5)	4 (4)	7 (4)
**Regular physical exercise** ‡						
Yes	453 (67)	69 (65)	128 (67)	63 (67)	79 (70)	114 (66)
No	198 (29)	36 (34)	53 (28)	27 (29)	29 (26)	53 (31)
Missing	25 (4)	1 (1)	10 (5)	4 (4)	5 (4)	5 (3)

BMI =  body mass index.

Note: out of total 680 Type 2 DM patients, 4 patients used other glucose lowering drugs and were excluded due to very less number.

δ Combination: Patients using more than one type of glucose-lowering drug or using combination tablets.

*Diet: very healthy (high amount of fruit, vegetables, fish and low amount of saturated fat), reasonably healthy (moderately high amount of fruit, vegetables, fish and saturated fat) or unhealthy (low amount of fruit, vegetables, fish and high amount of saturated fat).

† Alcohol intake: High (above the recommended limit of ≤14/≤21 weekly drinks for women/men) or low (within the recommended limit).

‡ Regular exercise: participation in leisure sports or other regular physical activity (yes/no).

**Table 5:** Age- and gender-standardized prevalence differences (PDs) of lifestyle factors according to glucose-lowering drugs.

	Metformin	Combinationδ	Sulfonylureas	Insulin and analogues	No glucose-lowering drug
	PD% (95% CI)	PD% (95% CI)	PD% (95% CI)	PD% (95% CI)	PD% (95% CI)
**BMI (kg/m^2^)**					
<18.5	Ref. (1.0)	–	–	–	–
18.5–24	Ref. (1.0)	2.8 (−5.0 to 10.6)	13.0 (3.0 to 23.0)	29.7 (18.8 to 40.6)	16.4 (7.4 to 25.4)
25–29	Ref. (1.0)	0.5 (−10.4 to 11.3)	6.9 (−5.8 to 19.6)	−7.0 (−18.7 to 4.7)	4.7 (−6.5 to 15.9)
≥30	Ref. (1.0)	−1.3 (−13.1 to 10.5)	−22.6 (−35.4 to −9.8)	−27.9 (−40.1 to −15.6)	−19.2 (−30.9 to −7.6)
**Diet** *					
Unhealthy	Ref. (1.0)	−1.6 (−8.5 to 5.3)	−0.6 (−8.4 to 7.2)	2.3 (−5.9 to 10.6)	2.8 (−4.7 to 10.3)
Reasonably healthy	Ref. (1.0)	9.6 (−2.2 to 21.3)	1.1 (−12.3 to 14.5)	−1.6 (−14.9 to 11.8)	3.3 (−8.8 to 15.5)
Very healthy	Ref. (1.0)	−9.8 (−20.4 to 0.8)	−4.7 (−17.1 to 7.6)	−2.3 (−14.4 to 9.8)	−10.0 (−20.8 to 0.9)
**Alcohol** †					
High	Ref. (1.0)	−3.8 (−9.4 to 1.9)	−3.5 (−9.6 to 2.6)	−1.2 (−7.7 to 5.3)	−2.3 (−8.2 to 3.6)
Low	Ref. (1.0)	6.2 (−1.5 to 13.8)	1.6 (−6.7 to 9.9)	2.9 (−5.8 to 11.6)	4.4 (−3.4 to 12.2)
**Smoking**					
Current	Ref. (1.0)	−5.2 (−14.6 to 4.2)	9.4 (−2.1 to 21.0)	2.9 (−8.5 to 14.2)	1.3 (−8.7 to 11.2)
Former	Ref. (1.0)	4.6 (−6.2 to 15.5)	−5.8 (−18.0 to 6.3)	−0.6 (−12.8 to 11.6)	−1.4 (−12.5 to 9.7)
Never	Ref. (1.0)	−3.7 (−14.1 to 6.7)	−5.6 (−17.6 to 6.3)	−1.9 (−13.8 to 10.1)	−3.4 (−14.1 to 7.3)
**Regular physical exercise** ‡					
Yes	Ref. (1.0)	1.8 (−9.5 to 13.1)	−3.0 (−15.8 to 9.9)	5.1 (−7.4 to 17.7)	1.9 (−9.7 to 13.4)
No	Ref. (1.0)	−6.5 (−17.6 to 4.7)	−2.7 (−15.4 to 9.9)	−9.2 (−21.3 to 3.0)	−4.0 (−15.4 to 7.4)

BMI =  body mass index, CI =  confidence interval.

δ Combination: Patients using more than one type of glucose-lowering drug or using combination tablets.

*Diet: very healthy (high amount of fruit, vegetables, fish and low amount of saturated fat), reasonably healthy (moderately high amount of fruit, vegetables, fish and saturated fat) or unhealthy (low amount of fruit, vegetables, fish and high amount of saturated fat).

† Alcohol intake: High (above the recommended limit of ≤14/≤21 weekly drinks for women/men) or low (within the recommended limit).

‡ Regular exercise: participation in leisure sports or other regular physical activity (yes/no).

#### Diet

In general, more metformin users reported eating reasonably healthy diet, compared with other Type 2 DM individuals. After standardizing for gender and age, the PRs for very healthy diet were decreased in users of combination therapy or no glucose-lowering drugs (PR: 0.7, 95% CI: 0.5–1.0) as compared with metformin therapy and were below 1 for other drug groups (*Table 6*).

**Table 6:** Age- and gender-standardized prevalence ratios (PRs) of lifestyle factors according to glucose-lowering drugs.

	Metformin	Combinationδ	Sulfonylureas	Insulin and analogues	No glucose-lowering drug
	PR (95% CI)	PR (95% CI)	PR (95% CI)	PR (95% CI)	PR (95% CI)
**BMI (kg/m^2^)**					
<18.5	Ref. (1.0)	–	–	–	–
18.5–24	Ref. (1.0)	1.3 (0.7 to 2.4)	2.2 (1.2 to 4.0)	3.6 (2.0 to 6.5)	2.5 (1.4 to 4.4)
25–29	Ref. (1.0)	1.0 (0.7 to 1.4)	1.2 (0.9 to 1.7)	0.8 (0.5 to 1.2)	1.1 (0.8 to 1.6)
≥30	Ref. (1.0)	1.0 (0.8 to 1.2)	0.5 (0.4 to 0.8)	0.4 (0.3 to 0.7)	0.6 (0.5 to 0.8)
**Diet** *					
Unhealthy	Ref. (1.0)	0.8 (0.4 to 1.8)	0.9 (0.4 to 2.1)	1.2 (0.6 to 2.6)	1.3 (0.6 to 2.6)
Reasonably healthy	Ref. (1.0)	1.2 (1.0 to 1.4)	1.0 (0.8 to 1.3)	1.0 (0.8 to 1.2)	1.1 (0.9 to 1.3)
Very healthy	Ref. (1.0)	0.7 (0.5 to 1.0)	0.9 (0.6 to 1.3)	0.9 (0.6 to 1.4)	0.7 (0.5 to 1.0)
**Alcohol** †					
High	Ref. (1.0)	0.5 (0.2 to 1.3)	0.5 (0.2 to 1.7)	0.8 (0.3 to 2.3)	0.7 (0.3 to 1.8)
Low	Ref. (1.0)	1.1 (1.0 to 1.2)	1.0 (0.9 to 1.1)	1.0 (0.9 to 1.1)	1.1 (1.0 to 1.2)
**Smoking**					
Current	Ref. (1.0)	0.8 (0.5 to 1.2)	1.4 (0.9 to 2.1)	1.1 (0.7 to 1.8)	1.1 (0.7 to 1.6)
Former	Ref. (1.0)	1.1 (0.8 to 1.6)	0.8 (0.6 to 1.2)	1.0 (0.7 to 1.4)	1.0 (0.7 to 1.4)
Never	Ref. (1.0)	0.9 (0.7 to 1.2)	0.9 (0.6 to 1.2)	1.0 (0.7 to 1.3)	0.9 (0.7 to 1.2)
**Regular physical exercise** ‡					
Yes	Ref. (1.0)	1.0 (0.9 to 1.2)	1.0 (0.8 to 1.2)	1.1 (0.9 to 1.3)	1.0 (0.9 to 1.2)
No	Ref. (1.0)	0.8 (0.6 to 1.2)	0.9 (0.6 to 1.4)	0.7 (0.5 to 1.1)	0.9 (0.6 to 1.3)

BMI =  body mass index, CI =  confidence interval, Ref =  reference.

δ Combination: Patients using more than one type of glucose-lowering drug or using combination tablets.

*Diet: very healthy (high amount of fruit, vegetables, fish and low amount of saturated fat), reasonably healthy (moderately high amount of fruit, vegetables, fish and saturated fat) or unhealthy (low amount of fruit, vegetables, fish and high amount of saturated fat).

† Alcohol intake: High (above the recommended limit of ≤14/≤21 weekly drinks for women/men) or low (within the recommended limit).

‡ Regular exercise: participation in leisure sports or other regular physical activity (yes/no).

#### Alcohol intake

Only 5% of all Type 2 DM patients reported high alcohol intake, and risk estimates associated with drugs were imprecise. However, standardized PRs for high alcohol intake were lower for all medication categories compared with metformin users, ranging from 0.5 (combination users and sulfonylurea users) to 0.8 (inulin and analogue users) (*Table 6*).

#### Smoking

The overall prevalence of current smokers ranged from 19% (combination therapy users) to 30% (sulfonylurea users). Standardized PRs for smoking were rather similar and close to 1 for most drug users, except for sulfonylurea users among whom smoking tended to be more prevalent (PR: 1.4, 95% CI: 0.9–2.1) compared to metformin users.

#### Physical exercise

The proportion with no regular physical exercise ranged from 26% (insulin and analogue users) to 34% (metformin users). After standardization, slightly smaller proportions without regular physical exercise were observed among all medication categories as compared with metformin users (PDs ranging from −2.7 to −9.2) (*Table 5*), with all PRs below 1 (*Table 6*).

## Discussion

### Main findings

This questionnaire-based public health survey contributes to the knowledge of how lifestyle choices differ according to use of glucose-lowering drugs. Patients with prevalent Type 2 DM were substantially more obese than individuals without diabetes, but otherwise reported to have a healthier lifestyle. Lifestyle characteristics appeared to differ between users of different glucose-lowering drugs. Metformin use was associated with adverse lifestyle factors, including higher obesity prevalence than other drugs user, and with a tendency towards higher alcohol intake and less physical exercise. Additionally, sulfonylurea use was associated with more current tobacco smoking, whereas insulin use was associated with less adverse lifestyle factors, including lowest BMI and highest physical exercise rates among the different glucose-lowering drug users.

### Comparison with other studies

Our results are partly in line with previous findings. Concerning obesity, Cichosz *et al*. reported higher BMI in 100 newly diagnosed Danish Type 2 DM patients compared to 100 age- and gender-matched population controls (30 kg/m^2^ vs. 26 kg/m^2^) [21]. Recently, 87% of newly diagnosed Danish Type 2 DM patients (median age at diagnosis: 59 years) were found to be either overweight or obese in the prospective DD2 study during 2010–2011, and 65% were obese [22]. Our age- and gender-standardized PR for obesity of 3.2 (95% CI: 2.8–3.6) associated with Type 2 DM versus other individuals is much in line with these figures, thus underlining the reliability of our results.

Results are conflicting from few available studies on other lifestyle habits comparing Type 2 DM and persons without diabetes. Magas *et al*. reported healthier nutritional habits among Type 2 DM patients compared with individuals without diabetes, corroborating the findings from our study [23]. In contrast, Murray *et al*. found that patients with Type 2 DM had lower dietary quality than general population controls as assessed by the Healthy Diet Indicator [24]. Concerning physical activity, we found increased self-reported levels among Type 2 DM patients, which is against the study by Cichosz *et al*. who reported less time spent on moderate to vigorous physical activity among Type 2 DM patients compared with controls without diabetes (34 minutes vs. 62 minutes) [21]. The discrepancies may be related to selection of hospital-based patients with relatively severe Type 2 DM, and/or recruitment of relatively healthy controls, in previous studies as compared with our population-based survey. Alternatively, our study may be hampered by potential information bias among Type 2 DM individuals, as e.g. physical activity was measured by questionnaires whereas Cichosz *et al*. measured it with electronic devices [21]. Finally, when comparing different Type 2 DM populations it must be kept in mind that population levels of obesity, smoking, alcohol intake etc. vary substantially between European countries [25], which is likely to affect proportions of unhealthy lifestyle habits in people with Type 2 DM as well.

Our findings corroborate previous findings of metformin users having the highest obesity prevalence and higher occurrence of several other unhealthy traits than other Type 2 DM patients do. Metformin is the recommended first-line drug for Type 2 DM patients with obesity [26], and many of our metformin users had a short history of diabetes with relatively little time for effective lifestyle changes. Our results are similar to the prevalence of obesity (54%) observed in metformin users in the United Kingdom Prospective Diabetes Study (UKPDS) [27], and slightly lower than the current obesity prevalence of about 60% in newly diagnosed Type 2 DM patients enrolled in the Danish DD2 study [22] of whom 59% are started with metformin monotherapy within the first year [28]. A large study based on the Swedish National Diabetes Register involving more than 51,000 Type 2 DM patients reported similar observation, with users of metformin-containing therapies having shortest duration of diabetes and higher mean BMI (30 to 32 kg/m^2^) than users of other oral glucose-lowering drugs or insulin (27 to 28 kg/m^2^) [29]. Of note, the Swedish study found much lower Type 2 DM smoking prevalence during 2004–2010 (14%) than observed in our data in 2006 (24%) [24]. In another Danish study of patients hospitalized with ischemic stroke, Horsdal *et al*. [9] reported a smoking prevalence of 27% in patients who had diabetes diagnosis, with patients on metformin therapy smoking most. An Italian study reported no substantial difference in the prevalence of smokers in metformin users (21%), sulfonylurea users (21%) and insulin users (22%) [30]. In contrast to our results, the study from Sweden found lower prevalence of smokers (12%) in sulfonylurea users as compared with metformin users (17%) [24], whereas the UKPDS reported higher prevalence of smokers in insulin users (39%) compared with metformin users (25%) [27].

Concerning alcohol intake, the distribution and pattern seen in our study was similar to findings in a recent Danish study of diabetes patients with ischemic stroke by Horsdal *et al*. [9], who reported 5.2% of all Type 2 DM patients and 7.5% of metformin users having higher alcohol consumption than recommended. Concerning physical exercise, we did not find any material difference between level of physical exercise among different medication users, which is in contrast to results reported by Ekstrom *et al*., who reported higher prevalence of physical activity (≥3 hrs/week) in patients using metformin (76%), compared to sulfonylurea users (70%) [29].

### Strengths and limitations

Strengths of this study include the availability of detailed lifestyle data for a large and randomly selected population-based sample included in a public health survey; and availability of additional information from independent population-based and highly valid medical registries [14], [16] for assessment of drug use and comorbidity, reducing the risk of recall or investigator bias. The response rate of 69% in the “How are you?” survey is rather high for a questionnaire study, and the 3.2% prevalence of Type 2 DM in our survey population is close to the national prevalence of 3.9% for diabetes in Denmark reported for the year of the survey in The Danish National Diabetes Register [31]. National public health surveys similar to the “How are you” are consecutively carried out every four years in all regions of Denmark, and are generally considered valid and of high quality [32]. As in most similar survey studies, people who choose to participate in a survey may have a different risk profile and may be in better health than those who decline. However, this probably applies for both Type 2 DM patients with different therapies and other individuals and is unlikely to bias the relative estimates in our study. Furthermore, as information on lifestyle factors was self-reported, we cannot rule out social desirability bias and it is possible that unhealthy lifestyles were underreported, which may potentially have led to an underestimation of unhealthy lifestyle in Type 2 DM. For instance, energy intake is underreported in persons with obesity and Type 2 DM [33], potentially due to external pressure to confirm to nutritional recommendations [34], and this might also hold true for other lifestyle risk behaviors. On the other hand, self-administered surveys are generally considered to be more suitable for sensitive questions on health status, as compared with direct interview surveys [35]. Additionally, in the questionnaire, the survey administrators aimed to minimise social desirability bias by asking several questions on the same topic, but from different perspectives. Another limitation of our study is its cross-sectional design, which implies uncertainty whether diagnosis of Type 2 DM or use of specific glucose-lowering drugs preceded lifestyle changes or vice versa. But, the main objective of our study was to describe differences in lifestyle behavior among people with and without Type 2 DM, and its association with current use of specific glucose-lowering drugs, and not to make inferences about causal mechanisms. Finally, inclusion of prevalent cases of Type 2 DM might have biased our results, however our estimates from sensitivity analyses did not change even after restricting the results among patients who were recently diagnosed.

Large database studies that examine the prognostic effect of Type 2 DM or glucose-lowering therapy will often be based on historical data and include a mixture of prevalent and incident Type 2 DM and drug users [36], [37]. Thus, we believe our data are useful and important to evaluate confounding in such studies, in which lifestyle factors are often not available.

## Conclusions

In conclusion, among participants in a large Danish population survey, patients with Type 2 DM were substantially more obese than other individuals, but otherwise reported a similar or healthier lifestyle after controlling for age and gender differences. Patients treated with metformin generally presented high BMI and tended to have less physical exercise and increased alcohol intake, whereas insulin users reported the lowest BMI and highest physical exercise. Patients on sulfonylureas tended to smoke more than other drug users. These estimates may be useful in external adjustment for unmeasured confounding in future database studies [10].

## Supporting Information

Appendix S1 World Health Organization International Classification of Diseases 8^th^ Edition (ICD-8) and 10^th^ Edition (ICD-10) codes, and Anatomical Therapeutical Chemical classification system (ATC) codes used in this study.
(DOCX)
