# Supplementary material for: Lifestyle Factors Associated with Type 2 Diabetes and Use of Different Glucose-Lowering Drugs: Cross-Sectional Study
Source: PLoS One. 2014 Nov 4;9(11):e111849. doi: 10.1371/journal.pone.0111849 (PMC4219789; doi:10.1371/journal.pone.0111849)
Supplement: Appendix S1 — World Health Organization International Classification of Diseases 8th Edition (ICD-8) and 10th Edition (ICD-10) codes, and Anatomical Therapeutical Chemical classification system (ATC) codes used in this study. (DOCX) [file pone.0111849.s001.docx]

##### Appendix S1

World Health Organization International Classification of Diseases 8^th^ Edition (ICD-8) and 10^th^ Edition (ICD-10) codes, and Anatomical Therapeutical Chemical classification system (ATC) codes used in this study

###### Type 2 diabetes mellitus

ICD-8: 249.x, 250.x.

ICD-10: E10.x, E11.x, E14.x, G63.2.x, H36.0, N08.3

###### Myocardial infarction

ICD-8: 410.x.

ICD-10: I21.x-I23.x

###### Congestive heart failure

ICD-8: 427.09-427.11, 427.19, 428.99, 782.49.

ICD-10: I50.x, I11.0, I13.0, I13.2

##### Glucose-lowering drugs

###### Insulin and analogues

ATC-codes: A10Ax

Metformin

ATC-codes: A10BAx

Sulfonylureas

ATC-codes: A10BBx

Other glucose-lowering drugs

ATC-codes: A10BFx, A10BGx, A10BHx, A10BXx

Combination tablets

ATC-codes: A10BDx
